# Supplementary material for: MetaRibo-Seq measures translation in microbiomes
Source: Nat Commun. 2020 Jun 29;11:3268. doi: 10.1038/s41467-020-17081-z (PMC7324362; doi:10.1038/s41467-020-17081-z)
Supplement: Supplementary file 10 — Supplementary Data 7 [file 41467_2020_17081_MOESM10_ESM.zip › File2/Confidence_VeryHigh_Taxonomy/156121_out.krona.html]

Javascript must be enabled to view this page.

members
magnitude
magnitudeUnassigned
count
unassigned
taxon
rank

156121\_out

14

superkingdom
14
2

1239
14
phylum

class
14
186801

order

SRS143417\_contig\_number\_8351
14
186802
1

family
1
31979

1
1485
genus

species

SRS014979\_contig\_number\_29308
1
1262828

186803
7
family

species
1898203
7

SRS014613\_contig\_number\_755SRS023715\_contig\_number\_20739SRS024663\_contig\_number\_8926SRS047433\_contig\_number\_9903SRS052697\_contig\_number\_contig-100\_4691.46968SRS065504\_contig\_number\_37858SRS148424\_contig\_number\_11733

5
1897043

SRS012273\_contig\_number\_13778SRS014923\_contig\_number\_28608SRS019068\_contig\_number\_87626SRS020233\_contig\_number\_12476SRS097889\_contig\_number\_contig-100\_46379.91125
species
